# Neural substrates of anorexia nervosa patient’s deficits to decode emotional information

**DOI:** 10.1007/s40519-020-00900-z

**Published:** 2020-05-01

**Authors:** Dorothée Lulé, Sabine Müller, Anne-Katharina Fladung, Ingo Uttner, Ulrike M. E . Schulze

**Affiliations:** 1 Department of Neurology, University of Ulm, Oberer Eselsberg 45, 89081 Ulm, Germany; 2 Department of Child and Adolescent Psychiatry/Psychotherapy, University of Ulm, Steinhövelstrasse 5, 89075 Ulm, Germany; 3 Institute of Psychology, Clinical Psychology and Psychotherapy, University of Hamburg, von-Melle-Park 5, 20146 Hamburg, Germany; 4 Department of Child and Adolescent Psychiatry, Centre for Psychiatry Calw, Bunsenstraße 120, 71032 Böblingen, Germany

**Keywords:** Anorexia nervosa, Emotion recognition, Anxiety, Depression, Alexithymia

## Abstract

**Purpose:**

The aim of the study was to define specific substrates of pathological behaviour patterns by analysing cortical activity using functional magnetic resonance imaging (fMRI) during an emotional processing task.

**Methods:**

In a sample of *N* = 11 adolescent patients with AN (16.36 years, SD ± 1.36) and *N* = 11 age-matched controls, we performed a functional MRI study to detect BOLD signal changes in a 3 T MRI scanner while presenting emotional facial stimuli.

**Results:**

Young people with AN presented with a generally reduced cortical activation pattern in key areas of emotion recognition for happy and fearful faces. Areas essential for control of social behaviour were associated with symptoms of depression.

**Conclusion:**

Obviously, there are already indications of cortical patterns in young affected persons, which indicate a changed emotional reaction to potentially aversive stimuli in the sense of a changed top-down process of emotion avoidance. Thus, the current study provides further evidence that the disorder of anorexia nervosa is closely related to deficits in emotion processing in the early course of ontogenesis. Depressive symptoms might additionally trigger pathological behavior. Due to the small sample size, the data should be considered preliminary and require further validation.

**Level of evidence:**

Level of evidence III: case–control study.

## Introduction

The avoidance of both, the experience and expression of strong emotions, is typically observed in individuals with anorexia nervosa, and researchers claim that it is an important issue in the emergence and maintenance of the disease [1]. Accordingly, individuals with AN seem to show altered emotional processing of visually presented faces and report higher alexithymia (i.e., a lower capability to identify and express their emotions) [2] than healthy individuals. Despite the probably crucial role of aberrant emotion processing in anorexia nervosa, research on its neural pathways is sparse: to our knowledge, only three fMRI studies on facial emotion perception in AN patients have been published so far, all of them examining adult subjects [3]. Findings are controversial, but overall indicating that neural correlates of facial emotion perception might be different in AN patients compared to healthy controls. To date, no fMRI studies on neural correlates of facial emotion perception in adolescents have been published.

In a previous study, we provided evidence that adolescents with AN present with recognition deficits of aversive stimuli such as disgust, whereas the positive emotion of happiness in faces was more easily recognized [4]. Emotion recognition deficits were with psychopathology, e.g., depressive symptoms. The aim of our current study was to determine cortical substrates of these emotion processing deficits in the previously reported adolescent sample [4] as evidence for early changes in anorexia nervosa.

## Materials and methods

### Participants

In total, *N* = 15 female adolescents between 14 and 18 years diagnosed with AN participated in the study (for further details see [4]). They were recruited through the Department of Child and Adolescent Psychiatry/Psychotherapy of Ulm University. In addition, 15 female healthy comparison subjects, who were group-matched for age, handedness, and education level, were recruited in state schools in Ulm. For all participants, prerequisite was a stable physical condition that allowed them to take part in such a challenging study. Exclusion criteria were: suicidal tendencies, substance abuse and/or dependence, cardiovascular diseases, epilepsy, Tourette syndrome, brain diseases, and endocrine diseases (e.g., hyperthyroidism). Additional exclusion criteria for healthy controls (HC) were eating and other psychiatric disorders. Due to missing data, four participants from each group were excluded from analysis, respectively, leaving *N* = 11 AN patients for the final analysis.

## Measures

### General assessment

All patients received a comprehensive clinical examination including medical and family history, neurological and psychiatric assessments and routine blood analysis to exclude physical illnesses apart from AN as reported previously (Table 1) [4]. Following clinical examination, participants performed fMRI scanning, followed by the FEEL test outside the MRI scanner [see 4] to determine subject’s ability to recognize facially expressed emotions as previously described (Table 1) [4]. To minimize influences of circadian rhythm, patients and controls were asked to perform the tests at the same daytime.

**Table 1:** Demographics and psychopathology of AN patients compared to controls

	Anorexia patients		Healthy controls		t test	
	Mean	SD	Mean	SD	*T*/*U*/*F*	*P*
Age	16.4	1.4	16.5	0.9	(*F*) 0.3	0.62
BMI	17.6	0.9	21.4	1.7	(*F*) 46.5	< 0.001**
HAWIK	102.3	9.4	101.6	5.8	(*T*) 0.2	0.83
TAS	49.7	13.6	39.7	7.4	(*T*) 2.1	0.05*
EDI	20.1	12.6	3.9	2.9	(*U*) 17.0	0.003**
EDE-Q	2.5	1.94	0.2	0.2	(*T*) 4.0	0.004**
STAI state	44.5	14.03	31.8	7.0	(*T*) 2.7	0.02*
STAI trait	47.9	11.01	32.6	8.1	(*T*) 3.7	0.001**
BDI	265.7	70.3	185.7	30.2	(*T*) 4.2	0.002**
FEEL total	34.4	4.1	35.4	4.6	(*T*) − 0.5	0.61
FEEL time	1238.8	355.6	1581.6	548.8	(*U*) 29.0	0.04*
FEEL happiness	7.0	0	6.8	0.4	(*U*) 49.5	0.15
FEEL surprise	5.8	1.25	6.3	1.1	(*U*) 45.5	0.33
FEEL disgust	4.8	1.6	5.9	1.3	(*U*) 36.0	0.12
FEEL anger	6.7	0.5	6.6	0.5	(*U*) 49.5	0.48
FEEL sadness	6.1	1.8	6.3	1.6	(*U*) 47.0	0.40
FEEL fear	4.6	1.5	4.2	2.1	(*T*) 0.6	0.56

A threshold of *p < 0.05 and **p < 0.001 (two-tailed) was used for statistical interference

Eating disorder psychopathology was assessed by the Eating Disorder Questionnaire (EDE-Q) and the Eating Disorder Inventory (EDI 2) for adolescents and adults. Depression was inquired by means of the Beck Depression Inventory II (BDI II). Total score (maximum 42 points) and the average response time per stimulus in the whole test is given. The 42 points are calculated by multiplying the six basic emotions and a maximum score per emotion of seven points. In addition, it can be determined which emotion was confused with which other emotion

*HAWIK* Hamburg–Wechsler-Intelligenztest for children—third edition (HAWIK-III), *TAS* Toronto alexithymia scale, *STAI* State and trait anxiety, *FEEL* facial expression of emotions test

## fMRI scanning

A functional MRI was performed on a 3 T Siemens Magnetom Allegra (Siemens, Erlangen, Germany). *T*2*-weighted images sensitive for Blood Oxygen Level Dependent (BOLD) contrasts were acquired every 2.14 s (repetition time, TR) with a voxel size of 3.6 × 3.6 × 3.2 mm^3^. The echo time (TE) was 32 ms and the flip angle (FA) was 90. Thirty-six slices were acquired in interleaved descending direction (Field of View = 200 mm, slice thickness = 3.2 mm, 25% interslice gap). Each of the three sessions contained 198 image volumes, resulting in a scan time of 7 min 8 s per session. The transversal slices were oriented along the AC/PC line. In addition, high-resolution *T*1-weighted anatomical images of the brain (TR = 2080 ms, TE = 3.93 ms, FA = 12, voxel size = 1 × 1 × 1 mm^3^) were obtained.

## fMRI design

An event-related design was applied to the paradigm presented during fMRI measurement. It was developed in order to get optimized signal-to-noise ratio. The stimuli are part of the original version of the Japanese and Caucasian Facial Expressions of Emotion stimulus set (JACFEE). In total, 191 pictures depicting 180 expressions of six emotions (happiness, sadness, anger, fear, surprise and disgust) of 60 individuals (half of them were of Caucasian and half of Japanese origin and gender distribution was even) were presented via video goggles during fMRI measurement [see 4]. Every individual was seen on three pictures, showing one emotion with an intensity of 60, 80 or 100%. Ten pictures of neutral faces were also included. One grey picture was generated as an average of all faces as a visual non-facial stimulus. Participants were presented 78 trials of pictures in total, divided into three runs of 26 trials of pictures each. One trial consisted of three different faces showing the same emotion with the same intensity, each shown for one second and divided by a fixation cross shown for one second. The fixation cross at the end of each trial appeared for a random time period (8–12 s). One trial took between 13 and 17 s in total. Nine trials of the six emotions and 12 trials of the neutral face and the grey picture appeared, respectively. The intensities of the depicted emotions were evenly distributed. The order of the stimulus presentation was pseudorandomized.

The instruction was to pay attention to all emotions displayed on faces. To control for attention, a photo of a woman whose face was not part of the item pool was presented at the beginning of each run, and participants were instructed to count how often she appeared in the following run. Following, a fixation cross appeared for a random time period, and the presentation of the stimuli began.

### Statistical analysis

#### Behavioral and clinical data

Questionnaire and test data were analysed using the Statistical Package for Social Sciences-24 (IBM® SPSS version 24.0). Statistical Parametric Mapping- 12 (Functional Imaging Laboratory, Institute of Neurology, UCL, London, UK) was used to conduct fMRI data analysis, running with Matlab R2016a (Release 2011b, The MathWorks, Inc., Natick, Massachusetts, United States). Analyses of self-report and behavioral data have been presented previously [4].

### fMRI data

The first five volumes of the functional run were discarded. Functional images were corrected for slice acquisition temporal onset differences. Subsequently, spatial realignment was performed on each fMRI data series by aligning all scans from each run with the first scan of the first run and aligning the images within runs with the mean of the images after the first realignment, in order to correct for residual interscan movement artifacts. Time series were normalized to the standard Montreal Neurological Institute *T*1 template (voxel size: 3.6 × 3.6 × 3.2 mm^3^) and spatially smoothed using a Gaussian kernel with a Full Width at Half Maximum (FWHM) of 8 mm. The tool ArtRepair was used for motion artifact correction: Outliers were selected by large variations in average global intensity or excessive scan-to-scan motion, and outlier volumes were interpolated.

For each subject, a first-level analysis was applied separately for each run, including the following condition types: all faces, disgust, happiness, surprise, anger, sadness, fear and neutral faces. Regressors were defined with onsets at the time of appearance of the corresponding event and convolved with the canonical hemodynamic response function. For the emotion conditions (disgust, happiness, surprise, anger, sadness, fear), the emotions’ intensities (60%, 80%, 100%) were included in the model. Six additional regressors were added in order to model the movement- and rotation parameters as confounds. For each subject, contrast images were calculated separately for each emotion condition compared with visual non-facial stimulus.

The individual contrast images were entered into separate second-level analysis to identify predictive voxels across subjects. Group activation maps were calculated using one sample *t* tests. Group comparisons were performed via two sample *t* tests. Regression was performed to test the null hypothesis of zero correlation between the series of scans and patient’s depressiveness (BDI).

Due to the explorative nature of the study and the small sample size, a cluster-wise corrected (50 voxels) with *p* < 0.05 was performed. Activation peaks were assigned to brain regions using probabilistic cytoarchitectonic mapping with the help of the SPM toolbox Anatomy, and via the SPM toolbox WFU Pickatlas. The WFU Pickatlas was further used to assign peaks to Brodmann Areas.

## Results

### Group differences in brain responses

Compared to the HC group, patients with AN showed a significantly reduced activation of the left midcingulate cortex (MCC; BA 31) during the processing of faces. There was no brain area that showed stronger activation in the AN group than in the HC group.

In response to happy faces, patients with AN revealed a significantly reduced activation of the right fusiform gyrus and a significantly reduced activation centred in the right caudate nucleus, extending to the right thalamus compared to healthy controls.

In response to fearful faces patients with AN showed a significantly reduced activation in the superior medial gyrus (i.e., the medial part of the superior frontal gyrus), predominantly on the left side extending to both sides of the posterior medial frontal cortex (pMFC) compared to healthy controls. In addition, they exhibited significantly reduced activation in the right side of the inferior frontal gyrus (pars orbitalis, Table 2).

**Table 2:** MRI activation pattern in AN in response to faces with different emotional expressions

	Brain region	Side	Peak MNI			K voxels	*T* (df)	*P*
			x	y	Z			
Activation in response to faces with different emotional expressions in patients with anorexia nervosa and healthy controls								
All faces								
HC > AN	Middle cingulate cortex (BA31)	L	− 5	− 29	42	101	5.60 (20)	< 0.001
HC < AN	None							
Happy faces								
HC > AN	Fusiform gyrus (BA 37)	R	31	− 44	− 12	50	5.46 (20)	< 0.001
	Caudate nucleus, thalamus	R	17	− 4	16	51	4.88 (20)	< 0.001
HC < AN	None							
Fearful faces								
HC > AN	Superior medial gyrus	L	2	32	48	71	4.81 (20)	< 0.001
	Inferior frontal gyrus	R	38	28	− 6	71	4.53 (29)	< 0.001
HC < AN	None							
Correlation of BOLD response in fMRI with psychopathology in patients with anorexia nervosa								
All faces								
BDI ( − )	Orbital gyrus	L	− 5	50	− 3	53	7.19 (13)	< 0.001

Threshold for significance uncorrected *p* < 0.001 and cluster-wise uncorrected *p* < 0.05 (50 voxels)

*BA* Brodman area, *MNI* Montreal Neurological Institute, *AN* patients with anorexia nervosa, *HC* healthy controls, *BDI* Beck Depression Inventory

AN patients presented with no increased activation to any stimuli compared to controls. Whole brain analysis revealed no significant group difference to the contrasts disgust, surprise, anger, and sadness.

### Correlation of brain responses with comorbid psychopathology scores in AN

BOLD response in the orbital gyrus to facial stimuli independent of emotional valence significantly correlated negatively with BDI scores (Table 2).

## Discussion

Studies published during the past years suggest altered activation in regions related to the fronto-striatal and limbic circuits in anorexia nervosa, which are suspected to play an important role in the development and maintenance of the psychopathology of AN. Our data also suggest an overall altered cortical pattern of emotion processing in AN. During facial processing, AN patients presented with a reduced BOLD activity in middle cingulate gyrus which is a key area of information processing in unpleasant context and to control the negative impact of potentially aversive social contexts [5]. For unpleasant stimuli such as fear, we found a significantly reduced cortical activity in superior medial frontal lobe, an area, which is crucial for emotion regulation and mentalizing [6]. This pattern of reduced cortical activity in areas of emotional processing is in line with the reduced capacity to recognize negative emotional face expressions [4]. However, participants displayed reduced performance for the facial emotion of disgust on the behavioral level in our previous study, whereas a subsample of this cohort which was measured in the current fMRI study showed no reduced cortical activity for disgust in any cerebral area which might be a matter of statistical power. Reduced cerebral activity was seen only for faces in general and for specific emotions of fear and happiness. The lack of activity in key areas of emotion recognition and regulation might suggest an additional top-down mechanism for fear and happiness in faces: the reduced activity in emotion processing and regulation areas might be facilitated by an avoidance strategy of subjects during a visual presentation of potentially (aversive) but also pleasant stimuli of facial expressions. However, as we did not provide any oculomotor data to determine fixation strategies of subjects (although all subjects were instructed to fixate the faces) and subject numbers were too low to correct for multiple comparisons, the discussion remains speculative.

Increased depression scores were associated with reduced orbitofrontal activity, an area, which is crucial for control of socially appropriate behaviour in specific, for appropriate food intake [7]. Orbitofrontal activity has been associated with the evolution of depression later in life [8]. The more depressed the patients in the current study were (as measured by BDI score), the less the area controlling appropriate behaviour such as food intake was activated. As loss of appetite and reduced food intake are key features of an affective state disorder, the pathologically reduced food intake in AN might additionally be triggered by this orbitofrontal loop via comorbid depressive symptoms.

In conclusion, adolescents with AN display an altered pattern of cortical BOLD activation. This pattern is suggestive of a top-down avoidance pattern of facial stimuli which are easily recognized. Our data are in agreement with previous research point to a significant involvement of the striatal reward system in AN. Altogether, we provide evidence for a multisystem disorder, with altered cortical pathways of social-emotional information processing of faces, which might be associated with avoidance strategies in social situations [9]. This complex interplay of dysfunctional activity in respective areas may lead to comorbid psychopathology, which then drives maladaptive behaviours [10] very early in development as we hereby present these changes in adolescent subjects.

### What is already known on this subject?

Patients with anorexia nervosa often find it difficult—frequently in part due to comorbid symptoms such as alexithymia, depression and anxiety symptoms—to perceive and express their own (negative or strong) feelings. The often limited perception of the other person’s emotions (via facial expressions) reinforces AN, especially as it can to contribute social withdrawal, a significant prognostic factor. So far, the neuronal correlates of the recognition of facial emotions have not been sufficiently researched—especially in the group of adolescents.

### What does this study add?

Our results suggest a higher complexity in neuronal changes related to emotion processing in patients with anorexia nervosa than previously thought. However, based on the model of a “multisystem disorder”, an etiological connection between perception, psychopathology and altered behaviour can be created. Further exploration of this connection would be desirable because it could lead to a holistic understanding of AN in young people.
